# Rapid Dilatation of the Female Urethra for the Relief of Vesical Irritability

**Published:** 1878-06

**Authors:** 


Rapid Dilatation of the Female Urethra for the Relief of Vesical Irritability.—Mrs. L., a married laundress, aged 31 years, has been under treatment for some months for right latero-flexion, associated with chronic endometritis consequent upon subinvolution following abortion in the third month. Three months ago acute cystitis suddenly developed, characterized by alarming prostration, intense burning pain on micturition, and the passage of bloody and muco-purulent urine. The acute symptoms having subsided under treatment, the usual means of relief were tried in vain to control the chronic irritability that remained. By chemical and microscopical examination, numerous pus and epithelial cells, and some albumen were found at various times in the urine. Otherwise it was normal. Topical and constitutional measures conferred no relief whatever. Nausea and vomiting were frequent and distressing symptoms. Constipation of the most obstinate character had existed for months. The uterine trouble had been undergoing satisfactory improvement previous to the development of cystitis, but since that occurrence the failure of the patient's strength, and the intense pain caused by the introduction of the speculum, interdicted the application of the remedies adapted to the relief of the former. Her worst symptoms seem to have proceeded from the vesical irritability. Every hour during the night a spirt of urine, amounting to a teaspoonful, would constitute her most serious trouble. Again, suffering until the pent-up urine was voided through the catheter. The passage of a soft rubber catheter always gave severe pain.

Fully convinced of the inutility of persisting in the ordinary modes of treatment, I determined upon performing rapid dilatation of the urethra as forming the only hope of relief. Accordingly, on February 28th, having evacuated the bladder and brought the patient under the influence of ether, I cautiously introduced into the urethra the blades of a narrow pair of sequestrum forceps, and gently dilated the passage until it would admit my little finger. This dilatation was increased to an extent which admitted of the introduction with ease of my thumb or forefinger. Urine ran freely from the bladder during the dilatation, notwithstanding the previous catheterization. The mucous membrane of the bladder communicated to the touch a soft, velvety feel, and seemed perfectly free from disease. The cavity was normal in size. Hemorrhage was slight.

March 1st.—Passed a restless night. Suffered excruciating pain in the vesical neck after each act of micturition. Urine was passed frequently during the night without her control. Morphia failed to ease her entirely. This morning she says she feels somewhat better—makes water with less pain. Tongue dry, and covered with a brownish coat. Pulse 115. Temperature 102° F. To have quinia and iron, with generous diet. Bromide of potassium at night.

2d.—Harrassed with incessant vomiting. Pain in bowels. Pulse extremely feeble. Tongue dry. Micturition free, but accompanied with burning pain. No vesical tenesmus. Complains of abdominal pain. Ordered a blister to abdomen, to remain five hours, and be replaced with a hot poultice. Bismuth, gr. v, lactopeptine, gr. x, as required to control vomiting.

3d.—Blister relieved her very much. Vomiting and pain returned towards evening. To have morphia and potass. brom.

5th.—Bladder a little worse; retains urine.

6th.—From this date the vesical trouble gradually augmented. Nitrate of silver topically; irrigation followed by injections of acetate of lead and morphia; suppositories—in short, the customary modes of treatment failed utterly to give the first shadow of relief. Evacuation of urine was performed every hour or two, to the amount of about a drachm each time. Finally, retention became established, and with it great tenesmus and suffering, as before.

26th.—No therapeutic resource being left which promised any useful purpose, I determined again to dilate, and this time carry the stretching to the extent practiced by Teale. An anæsthetic having been administered, I dilated as before, introduced the right index finger, crowding it gradually up, and then withdrawing, introduced the indices of both hands, stretching the urethra and vesical neck to their utmost capacity consistent with safety, with a view to completely overcome the spasm of the sphincter, and render it powerless to produce subsequent closure. Some pain was experienced after recovery from the anæsthetic, but was slight compared with that following the previous operation. Immediate relief was obtained from the more urgent symptoms, the only symptom complained of after the operation being a slight burning sensation accompanying each act of micturition. This gradually became less, and her progress has been most gratifying.

At the present date, April 14th, the vesical irritability has entirely disappeared, and she states that her bladder is all right now, and the relief she has experienced cannot be expressed in words.

I regard this case as furnishing a crucial test of the efficacy of Teale's operation for the relief of the very distressing symptoms of irritable bladder. From previous recorded experience it appears that this condition, complicating uterine versions or flexions, has undergone little or no improvement from urethral dilatation, and in the present case I confess that I regarded the operation as holding out but slight promise of cure, and had recourse to it only as a dernier resort, after the complete failure of all other measures. It is possible that failure in some cases has been due to incomplete dilatation, as it was in my case after the first operation. It therefore seems proper to practice extreme dilatation before abandoning the operation as useless. The fullest distention consistent with safety appears to be reached when the channel has been enlarged to about an inch in diameter.—*N. Y. Med. Record.*

